# Viral Diagnostics in Plants Using Next Generation Sequencing: Computational Analysis in Practice

**DOI:** 10.3389/fpls.2017.01770

**Published:** 2017-10-24

**Authors:** Susan Jones, Amanda Baizan-Edge, Stuart MacFarlane, Lesley Torrance

**Affiliations:** ^1^Information and Computational Science Group, The James Hutton Institute, Dundee, United Kingdom; ^2^School of Biology, The University of St Andrews, St Andrews, United Kingdom; ^3^Cell and Molecular Science Group, The James Hutton Institute, Dundee, United Kingdom

**Keywords:** viral diagnostic, next generation sequencing (NGS), crop protection, food security, bioinformatics & computational biology

## Abstract

Viruses cause significant yield and quality losses in a wide variety of cultivated crops. Hence, the detection and identification of viruses is a crucial facet of successful crop production and of great significance in terms of world food security. Whilst the adoption of molecular techniques such as RT-PCR has increased the speed and accuracy of viral diagnostics, such techniques only allow the detection of known viruses, i.e., each test is specific to one or a small number of related viruses. Therefore, unknown viruses can be missed and testing can be slow and expensive if molecular tests are unavailable. Methods for simultaneous detection of multiple viruses have been developed, and (NGS) is now a principal focus of this area, as it enables unbiased and hypothesis-free testing of plant samples. The development of NGS protocols capable of detecting multiple known and emergent viruses present in infected material is proving to be a major advance for crops, nuclear stocks or imported plants and germplasm, in which disease symptoms are absent, unspecific or only triggered by multiple viruses. Researchers want to answer the question “how many different viruses are present in this crop plant?” without knowing what they are looking for: RNA-sequencing (RNA-seq) of plant material allows this question to be addressed. As well as needing efficient nucleic acid extraction and enrichment protocols, virus detection using RNA-seq requires fast and robust bioinformatics methods to enable host sequence removal and virus classification. In this review recent studies that use RNA-seq for virus detection in a variety of crop plants are discussed with specific emphasis on the computational methods implemented. The main features of a number of specific bioinformatics workflows developed for virus detection from NGS data are also outlined and possible reasons why these have not yet been widely adopted are discussed. The review concludes by discussing the future directions of this field, including the use of bioinformatics tools for virus detection deployed in analytical environments using cloud computing.

## Introduction

Rapid detection and identification of viruses in cultivated plants is very important for successful crop production. Viruses cause significant yield and quality losses in a wide variety of agricultural crops and have an important negative economic impact (Rybicki, 2015). All types of crops are susceptible to virus infection, including those cultivated for food, as ornamentals and for fuel. Examples include Potato virus Y (PVY) infection of potato and Turnip mosaic virus infection of oilseed rape. Classical infection symptoms, such as yellowing, mosaic and stunting are often not diagnostic, and can be absent or masked by other factors. It is also common for combinations of different viruses to be the trigger that leads to severe infection symptoms (Syller, 2012). With a rising international trade in seeds and stock plants and agricultural intensification, there is an increasing likelihood of new and emerging viruses becoming established (Massart et al., 2017). Hence, effective plant viral diagnosis is an essential tool to help deliver world food security.

**Figure 1:**
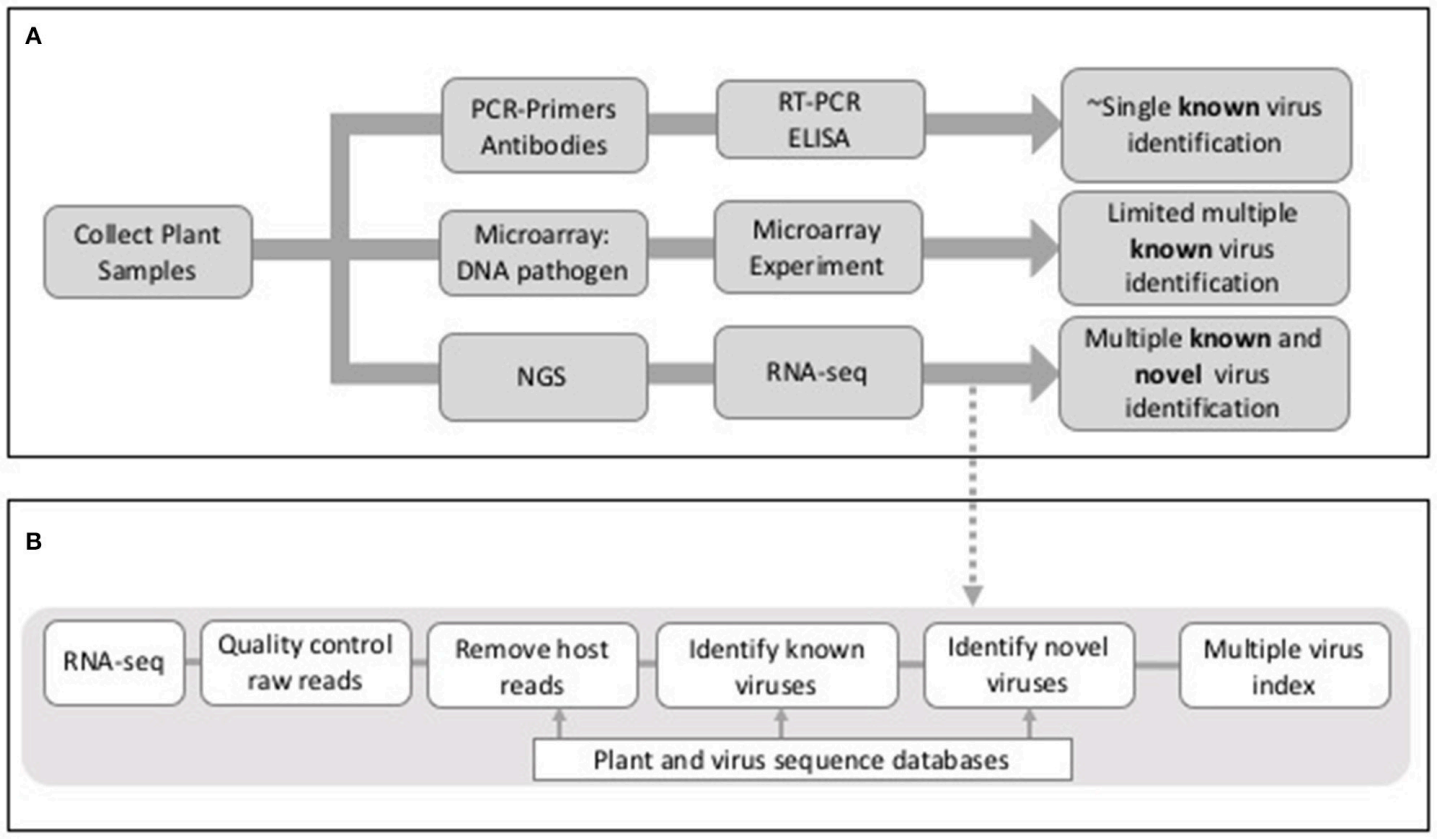
**(A)** Outline of outcomes from PCR, microarray, and NGS-sequence based approaches for virus detection in plants. **(B)** Outline of potential stages in an RNA-seq analysis workflow for virus detection in plants.

Whilst the adoption of molecular techniques such as reverse transcriptase-polymerase chain reaction (RT-PCR) has increased the speed and accuracy of viral disease diagnosis in crops, such techniques only allow the detection of known viruses, i.e., each test is specific to one or a small number of related viruses (Mumford et al., 2006). If such techniques are unavailable, or the virus is unknown, then disease diagnosis requires tests conducted using indicator plants in expensive glasshouses or the use of field indexing, both of which are laborious and slow. Methods for simultaneous detection of multiple viruses (multiplexed methods) have been developed, and next generation sequencing (NGS) is now a principal focus in this area (Boonham et al., 2014; Figure 1). The development of new techniques capable of detecting multiple viruses present is essential for when disease symptoms are absent, unspecific or triggered only when plants become infected by multiple viruses. In such instances, a specific single-pathogen diagnostic would not be able to identify the cause of disease in all outbreaks. The majority of plant viruses have RNA as their genetic material and those that have DNA genomes produce RNA transcripts. Hence the analysis of RNA sequences from plant samples is an effective method for virus detection. More recently the sequencing of total small RNAs (sRNAs) has also proved to be an effective method for virus detection (Wu et al., 2010). Whilst controversial in mammals, eukaryote small interfering RNAs (siRNAs) direct antiviral immunity through RNA interference and during this process virus-derived siRNAs are enriched in the host and can be selectively purified for sequencing (Wu et al., 2010).

The ability to conduct hypothesis-free viral testing of plants using NGS, presents both opportunities and challenges. Researchers want to answer the question “how many different viruses are present in this crop plant?” without knowing what they are looking for: RNA-sequencing of plant material allows this question to be addressed. A number of studies have recently been published that have used this approach to establish the cause of non-specific plant disease symptoms (e.g., Matsumura et al., 2017), to establish the viral load of native or imported plant stocks (e.g., Wylie et al., 2014) or to exemplify methods for re-analyzing existing RNA-seq datasets for virus detection purposes (e.g., Jo et al., 2015). Publications that attempt to establish the total viral load of a plant have generally been exploratory in nature, and the wider impact of NGS technology for virus detection in plants is only just emerging as the number of studies increases.

### Using RNA-seq for virus detection in plants

Whilst studies on virus identification by NGS sequencing are dominated by those using human clinical samples, those looking at viruses in plants have been slower to emerge. However, as previously stated, a number of papers have now been published that use RNA-sequencing analysis for hypothesis-free detection of viruses in a different crop plants (Coetzee et al., 2010; Kashif et al., 2012; Li et al., 2012; Wylie et al., 2014; Jo et al., 2015, 2016, 2017; Matsumura et al., 2017). All these papers describe analyses in agricultural crops, and show the need for methods that can identify multiple viruses, as viral co-infection is a consistent theme. Each study is described here, with an emphasis on the computational methods used to analyse the RNA-seq data for virus detection.

#### Garlic (*Alium sativum*)

The viral content of garlic has been analyzed using RNA-seq to make the case for using multiplex methods for virus detection in the context of plant quarantine systems (Wylie et al., 2014). Total RNA was extracted from leaves from garlic plants (both imported and native to Australia), amplified and sequenced using Illumina HiSeq 2000 technology. Two commercial software packages [Genius Pro (www.geneious.com) and CLC Genomics Workbench (www.clcbio.com)] were used to *de novo* assemble reads into contigs. Contigs with lengths >1,000 nucleotides were then aligned to the GenBank database (Benson et al., 2013) using Blastn and Blastx (Altschul et al., 1990). Contigs with one or more hits to a known virus within the top 100 Blast hits were then further classified. The raw reads were then mapped back to each contig representing putative viral sequences, and those with less than 10-fold coverage were removed. Open reading frames (ORFs) in contigs with no matches to viral sequences were identified, and the amino acid sequence of predicted large ORFS compared against the Conserved Domain Database (Marchler-Bauer et al., 2015). This study revealed that isolates of between 1 and 8 viruses were present in each cultivated garlic plant (*A. sativum*) and a single virus isolate was detected in one wild garlic plant (*A. vineale L*.). In total 41 virus isolates were identified including potyviruses (e.g., Leek yellow stripe virus), allexiviruses (e.g., Garlic virus D (GarVD) and carlaviruses (e.g., Shallot latent virus). This study was the first to obtain the complete genomes of two isolates of GarVD, and to show the presence of the potexvirus Asparagus virus 3 in wild garlic (which grows as a weed) in Australia.

#### Pepper (*Capsicum annuum*)

Multiple viral infections have also been identified in pepper plants (*Capsicum annuum*) using RNA-seq analysis (Jo et al., 2017). In this study two different pepper cultivars [Pusa Jwala (PJ) (susceptible) and Taiwan-2 (TW) (resistant)] were sequenced using HiSeq 2000 technology. The raw reads were *de novo* assembled using both Trinity (Grabherr et al., 2011) and Velvet (Zerbino and Birney, 2008) with Oasis (Schulz et al., 2012). The resulting contigs were compared to the RefSeq viral database (O'Leary et al., 2016) using MEGABLAST. This study compared the use of Trinity and Velvet for *de novo* assembly, and for these data Trinity was shown to be better, producing longer contig lengths, but Velvet was considered better for assemblies with low virus titre. The use of Trinity and Velvet to assemble contigs resulted in different combinations of viruses being identified for each assembler. However, eight viruses were common to all datasets, with Bell pepper endornavirus (BPEV), PepLCBV (Pepper leaf curl Bangladesh virus), and TVCV (Tobacco vein clearing virus) having the highest number of contigs matched. In addition this study identified a novel virus, Pepper Virus A (PepVA).

#### Pear (*Pyrus pyrifolia*)

Many fruit trees are known to be co-infected by multiple viruses, and the analysis of a pear (*Pyrus pyrifolia*) transcriptome helped to confirmed this (Jo et al., 2016). The study used publically available transcriptome libraries from 3 different studies, 2 mRNA-seq and 1 sRNA-seq to look at infection by Apple stem grooving virus (ASGV), most commonly identified in apples, pear and citrus trees. The pear transcriptome, sequenced during different developmental stages, was extracted from the Sequence Read Archive (SRA) (Leinonen et al., 2011) (SRA identifier: SRX532394). The raw reads were *de novo* assembled using Trinity (Grabherr et al., 2011) and the assembled contigs compared against reference viral genomes using MEGABLAST. This analysis revealed the presence of 5 viruses with read counts >5 which included ASGV, but also 3 additional viruses, Prunus virus T (PrVT), Apple green crinkle associated virus (AGCAV), and Apple stem pitting virus (ASPV). Additional reads were initially matched to Potato leaf roll virus, but on further analysis these contigs were identified as host sequences.

#### Grapevine (*Vitis vinifera*)

Two studies have looked at virus co-infection of grapevines; (Coetzee et al., 2010; Jo et al., 2015). In the first, lignified cane material was selected from vines (*Vitis vinifera*) in a merlot vineyard in South Africa (Coetzee et al., 2010). RNA-seq was conducted using Illumina Genome Analyzer technology to give paired-end reads. The reads were *de novo* assembled using Velvet (Zerbino and Birney, 2008) and the contigs compared to the NCBI non-redundant DNA and protein databases using Blast (Altschul et al., 1990) and classified. The viruses identified included Grapevine leafroll-associated virus 3 (GLRaV-3), Grapevine rupestris stem pitting-associated virus (GRSPaV) and Grape vine virus A (GVA). Grapevine virus E was also identified, which had not previously been reported in vineyards in South Africa, and in addition this study was the first to isolate and identify mycoviruses in grapevine phloem.

In later work the transcriptome of the grapevine cultivar Tannat, that had been sequenced in an earlier study (Da Silva et al., 2013), was extracted from the SRA and re-analyzed for the presence of viruses (Jo et al., 2015). The transcriptome was composed of multiple libraries from three different grapevine tissues; grain, skin and seed, and paired-end sequencing had been conducted using the HiSeq 1000 technology (Da Silva et al., 2013). *De-novo* assembly was conducted with the raw reads from each library using Trinity (Grabherr et al., 2011) and the contigs compared against virus reference genome sequences using Blast (Altschul et al., 1990). Across all the libraries the most prevalent viruses identified were Grapevine yellow speckle viroid 1 (GYSVd1), Grapevine pinot gris virus (GPGV), Hop stunt viroid (HSVd), and Grapevine leafroll-associated virus 2 (GLRaV2). The most prevalent virus was different in each library. This study also found that the distribution of some viruses varied between the different tissue types. Whilst 4 viruses, (GRSPaV-1, GPGV, GYSVd1, and HSVd), were identified in all 3 tissues, Oat blue dwarf virus (OBDV) and Potato virus S (PVS) were only identified in the seed tissues, and skin tissue was shown to have a higher prevalence of viruses in general than grain tissue.

#### Sweet potato (*Ipomoea batatas*)

Sweet potato (*Ipomoea batatas*) is known to be infected by more than 30 viruses (Clark et al., 2012) and this was investigated in a study which sequenced sRNAs from sweet potato in Honduras and Guatemala (Kashif et al., 2012). sRNAs were isolated from total RNA extracted from leaf material and sequenced using Illumina Genome Analyzer technology. Velvet (Zerbino and Birney, 2008) was used to assemble short reads and the contigs were compared against the NCBI non-redundant database using Blast (Altschul et al., 1990). The virus sequences identified were then used as references to make alignments of the short reads using MAQ (Li et al., 2008) and to make assemblies of individual viruses. This method enabled the simultaneous detection of three RNA viruses [Sweet potato chlorotic stunt virus strain WA (SPCSV-WA), Sweet potato feathery mottle virus (SPFMV-RC), and Sweet potato virus C (SPVC)], and two DNA viruses [Sweet Potato leaf curl Georgia virus (SPLCGV) and Sweet potato pakakuy virus strain B (SPPV-B)]. The study also showed that some plants were co-infected with more than one virus, and in specific cases this affected the severity of disease symptoms. For example, plants infected with SPPV-B developed leaf symptoms of varying severity, but when disease symptoms were severe SPCSV-WA was always present.

#### Orange fruit (*Citrus sinensis*)

Over 4 million orange trees (*Citrus sinensis*) have recently been lost from Citrus sudden death (CSD) disease in the Sao Paulo State of Brazil. It was thought that CSD was caused by a variant of the Citrus tristeza virus (CTV) and RNA-seq was performed on CSD-symptomatic and -asymptomatic plants to test this hypothesis (Matsumura et al., 2017). Sequencing was conducted using Illumina HiSeq 2000 technology and raw reads *de novo* assembled using the CLC Assembly Cell (CLC Bio-Qiagen) and Trinity (Grabherr et al., 2011). The contigs were mapped to the host genome and host contigs removed. Unmapped contigs were compared against the NCBI non-redundant (nr) virus protein database using Blastx (Altschul et al., 1990), and potential virus sequences were individually checked to confirm the virus classification. Contigs that shared high sequence identity with the same virus species were compared against the nucleotide NCBI nr virus nucleotide database using BLASTn to identify virus isolates. This methodology showed mixed infections that included CTV, Citrus sudden death-associated virus (CSDaV), Citrus endogenous pararetrovirus (CitPRV) and two putative novel viruses named as Citrus jingmen-like virus (CJLV), and Citrus virga-like virus (CVLV). This study was additionally able to differentiate two genotypes for both CTV and CSDaV, and indicated that one CSDaV genotype was associated with symptomatic plants.

#### Tomato (*Solanum lycopersicum*)

The final study discussed here is one that investigated viruses infecting tomatoes in the US and Mexico (Li et al., 2012). In this work sRNAs were sequenced from tomato plants with disease symptoms using the Genome Analyzer II technology. The raw reads were first aligned to the tomato genome using BWA (Li and Durbin, 2009) and un-aligned reads were aligned to the Genbank (Benson et al., 2013) virus collection again using BWA. The sRNA reads were also *de novo* assembled using Velvet (Zerbino and Birney, 2008), the sRNAs aligned back to the assembled contigs using BWA, and the base coverage at each position of the contigs calculated. The final contigs were compared against GenBank (Benson et al., 2013) nt and nr databases, respectively, using BLAST (Altschul et al., 1990). Contigs with significant similarity to known viruses were identified as candidate virus sequences. Using this method the complete genomes of six Pepino mosaic virus (PepMV) isolates and a Potato spindle tuber viroid (PSTVd) isolate were assembled. In addition, two strains of PepMV (EU and US1), present as a mixed infection, were differentially assembled into their respective genomes and a novel potyvirus was detected and its full genome assembled.

There are a number of common threads that are highlighted by these studies; (a) co-infection of individual plants with more than one virus, (b) the identification of viruses in asymptomatic plants, (c) different viruses or levels of viruses associated with different parts of the plant, (d) different analysis tools giving rise to different viruses being detected, (e) the identification of novel viruses and/or novel virus isolates, and (f) identification of viruses in specific geographical regions or in host species where they have not been observed previously. As summarized, these studies were conducted using different virus detection workflows that included different algorithms, tools and databases. The main elements of many of the methods included (a) quality control of the raw reads, (b) assembly of raw reads into contigs, (c) the removal of host sequences by alignment to a host genome and (d) identification of viral reads by mapping to a virus database (Figure 1). Whilst the same short read mapper, alignment and assembly tools were shared by many studies [Bowtie2; Langmead, 2013, Trinity Grabherr et al., 2011, Velvet (Zerbino and Birney, 2008), Oases (Schulz et al., 2012), Blast (Altschul et al., 1990)] they were applied with different parameters and thresholds, specific for the RNA-seq data being analyzed.

### Bioinformatics tools for virus detection using RNA-seq data

The need to achieve the types of analyses described in the previous section has given rise to a number of bioinformatics tools designed to complete the analysis in a workflow. Recent methods (including those for the analysis of RNA-seq and sRNA-seq) are summarized in Table 1, where it is clear that the predominant focus of the majority of these tools has been the identification of viruses in human clinical samples. This is an easier problem to address, as the availability of the human genome allows for rapid subtraction of host sequence, and human virus data predominates in sequence databases, making multiple virus detection possible. Comparably, the genomes of many crops are unknown or incomplete, and plant virus sequences are poorly represented in databases.

**Table 1:** Bioinformatics tools for the identification of viruses in RNA-sequence samples.

**Tool**	**Reference**		**Strategy**	**Benchmarking**	**Seq Input**	**Availability**
VirFind	Ho and Tzanetakis, 2014	Web based tool that maps and removes host reads, gives taxonomic information for virus reads	Quality Control Mapping to host: Bowtie2 (Langmead, 2013) Assembly of non-host reads: Velvet Contigs compared with GenBank using Blastx Unmapped reads translated and compared against NCBI conserved domain database (Marchler-Bauer et al., 2015)	38 Plant samples from 19 species	sRNA mRNA	Web based tool with GUI http://virfind.org
Taxonomer	Flygare et al., 2016	Fast web-based metagenomics analysis tool based on *k-mer* profiling	Comprised of 4 modules Binner: compares reads to reference *k-mer* database [based on 21-kmers created using Kanalyze (Audano and Vannberg, 2014)] assigning to broad taxa (human, virus, bacteria) Classifier: exact *k-mer* matching within taxonomic bins against *k-mer* databases created from UniRef datasets (Suzek et al., 2015) (e.g., viral UniRef90) Protonomer: further classification in protein space AfterBurner: discovery of novel taxa	Human	mRNA	Webserver: https://www.taxonomer.com/
VSD toolkit	Barrero et al., 2017	Modules and workflows in the Yabi analytical environment for identification of viral sequences in plants	Quality Control *De novo* assembly: SPAdes (Bankevich et al., 2012) Overlapping contigs merged Contigs >40 nt aligned to plant, virus and viroid Genbank databases using Blastn and Blastx Unmapped contigs filtered and analyzed to identify putative circular viroids	21 Plant genomes	sRNA	Source code available to use with Yabi (Hunter et al., 2012) https://github.com/muccg/yabi
Metavisitor	Carissimo et al., 2017	Modular tools and workflows within the Galaxy analytical environment, designed for detection and reconstruction of viral genomes	Quality control Mapping to host, symbionts and parasites: Bowtie2 Unmapped reads assembled using Velvet + Oases (Schulz et al., 2012) or Trinity Contigs compared to Genbank virus database using Blastx and Blastn Blast guided scaffolding of selected virus sequences	Human Drosphila Mosquito	mRNA	Source code available to use within Galaxy (Afgan et al., 2016). Galaxy Toolshed: suite_metavisitor_1_2 Galaxy instance: https://mississippi.snv.jussieu.fr/
VIP	Li et al., 2016	An integrated pipeline for metagenomics of virus identification and discovery	Quality Control Mapping to host: Bowtie2 Fast Mode: reads mapped to Virus Pathogen Resource (ViPR) (Pickett et al., 2012) and Influenza Database (Squires et al., 2012) Sense mode: reads mapped to virus RefSeq (O'Leary et al., 2016) nucleotide Unmapped reads mapped to RefSeq protein: RAPSearch (Zhao et al., 2012) Options for *de novo* assembly with Velvet-Oases	Human	mRNA	Local Installation. Code available at https://github.com/keylabivdc/VIP
ViromeScan	Rampelli et al., 2016	Tool for metagenomics viral community profiling	Mapping to built-in databases (includes plants): Bowtie2 Mapped reads processed for quality Human Best Match Tagger (BMTagger) (Agarwala and Aleksandr, 2011) used to screen out human and bacterial sequences Screened reads re-mapped to virus database: Bowtie2	Human	mRNA	Local installation. Code available at http://sourceforge.net/projects/viromescan
VirusHunter	Zhao et al., 2013	Data analysis pipeline for novel virus identification from Roche 454 sequencers and other long read platforms	Similar reads clustered using CD-Hit (Li and Godzik, 2006) and longest sequence used as representative Repeat regions masked with Repeat Masker (Smit et al., 2013) Mapping to host (default: Human): Blastn Unmapped sequences aligned to NCBI nucleotide database and classified into taxonomies Unmapped sequences mapped to NCBI nr databases using Blastx	BHK (hamster) cell culture infected with viruses	mRNA	http://pathology.wustl.edu/VirusHunter/Code available upon request for local installation
ezVIR	Petty et al., 2014	Bioinformatics pipeline to evaluate spectrum of known human viruses	Mapping to host (Human genome): Bowtie2 Nonhost mapped to custom virus database: Bowtie2 Additional analysis on specific mapped classes provides targeted strain classification	Human	mRNA	Local installation. Code available: http://cegg.unige.ch/ezvir
Virus Detect	Zheng et al., 2017	Bioinformatics pipeline to analyse sRNA datasets for both known and novel virus identification	Maps reads to virus reference sequences: BWA Mapped reads assembled using references Mapped reads *de novo* assembled using Velvet Reference assemblies and *de novo* assemblies pooled and redundant sequences removed Contigs compared to virus reference nulcotides: Blastn Unmatched contigs matched to virus reference Blastx	Plants (Potato)	sRNA	Webserver: http://bioinfo.bti.cornell.edu/tool/VirusDetect
VirusFinder	Wang et al., 2013	Software for detection of viruses and their host integration sites	Mapping to host (Human genome): Bowtie2 Non-host reads mapped one of two virus databases (Hirahata et al., 2007; Bhaduri et al., 2012): Bowtie2 Mapped reads *de novo* assembled: Trinity Contigs mapped to virus database and human genome For specific virus of interest host integration sites are identified using BWA (Li and Durbin, 2009)	Human	RNA-seq WGS Targeted	https://bioinfo.uth.edu/VirusFinder/

*References for databases and software with multiple entries in table: Genbank (Benson et al., 2013), Blastx and Blastn (Altschul et al., 1990), Velvet (Zerbino and Birney, 2008), Bowtie2 (Langmead, 2013), Trinity (Grabherr et al., 2011)*.

Any virus detection workflow needs to be capable of: (1) uploading raw sequence reads (comprising both host and virus) from a sequencing platform, (2) conducting quality control measures on raw data files, including trimming of poor quality reads and adaptor sequences, (3) identifying and removing host reads, (4) identifying known viruses and ideally (5) providing a method for the identification of novel viruses and/or strains (Figure 1). As described previously the main strategy for achieving this is through a combination of assembly and mapping (Table 1). The one exception to this is Taxonomer (Flygare et al., 2016) which takes advantage of a new development in this field; *k-mer* profiling (Flygare et al., 2016). Nucleotide sequences can be treated as character strings and divided into multiple substrings of length *k*. In this way, a sequence can be represented by *k-mer profiles*, and these profiles can be compared to reference databases for taxonomic assignment. *K-mer* based methods are much faster than alignment methods and they have successfully been used in the identification of viral haplotypes (Malhotra and Sowdhamini, 2013). As with all bioinformatics tools, when published, the virus detection workflows outlined in Table 1 have been tested on be-spoke datasets using ideal computing environments and with knowledge of optimal parameters. Hence publications lay claim to methods being faster or better than others, when robust benchmarking has often not been conducted.

While it is possible to sequence the transcriptome of an infected plant for < £500 per sample, the real bottleneck (and cost) comes in analyzing the data in a meaningful way. In small research groups without a bioinformatician or access to a bioinformatics core facility, sequence analysis is often conducted by the non-expert using online resources. Whilst the latest published method might offer “better” virus detection, it is of little use to the non-expert if the software requires local installation, knowledge of the Linux operating system, is dependent upon the installation of additional programs or modules or the installation of a separate analytical environment. So we asked the question; of those tools in Table 1, which ones can actually be used by the non-expert with RNA-seq data from an infected plant?

### Virus detection in practice

Those tools providing online access, not requiring local installation of software or analysis environments, and with the potential to analyse plant samples are Taxonomer (Flygare et al., 2016), VirusDetect (Zheng et al., 2017) and Virfind (Ho and Tzanetakis, 2014). Only the first two provide a true web interface that includes file upload, analysis and provision of results interactively. VirFind (Ho and Tzanetakis, 2014) provides a web interface for the submission of a form that details the analysis and files to be upload. File upload is through FTP and results are provided via email, usually after 2–3 days for large datasets. Hence, we chose to conduct a simple test of Taxonomer (Flygare et al., 2016) and VirusDetect (Zheng et al., 2017) on 3 datasets derived from studies with data deposited in the SRA (Leinonen et al., 2011). The RNA-seq datasets used were from pear, pepper and grapevine (Table 2), and the aim of the test was to evaluate if automatic virus detection was possible and comparable to the results described in the original studies of these datasets.

**Table 2:** RNA-seq datasets used to test two automated virus detection tools.

**Organism**	**SRA ID**	**Data type**	**Viruses detected in original analysis**
Pear (*Pyrus pyrifolia*)	SRR1269627	RNA-seq, SE	ASGV, AGCAV, ASPV, PrVT (Jo et al., 2016)
Pepper (*Capsicum annuum*)	SRR1123893	RNA-seq, PE	ALPV, BPEV, cgLCuV, CYVMVA, PepLCB, PepLCBV, PeSV, ToLCRnV, ToLCBDB, ToLCJoV, GaILV, TolCGV, TVCV (Jo et al., 2017)
Grape Vine (*Vitis vinifera*)	SRR3680863	sRNA-seq, SE	GRSPaV, GVB, GFkV, GLRaV-3, HSVd (Barrero et al., 2017)

*The specific sequence datasets used are indicated by their SRR number in the SRA (Leinonen et al., 2011). SE, Single-end; PE, Paired-end*.

*Pear: Prunus virus T (PrVT), Apple green crinkle associated virus (AGCAV), Apple stem grooving virus (ASGV), Apple stem pitting virus (ASPV). Pepper: Aphid lethal paralysis virus (ALPV), Pepper leaf curl Bangladesh virus (PepLCBV), Pea streak virus (PeSV), Pepper leaf curl virus betasatellite (PepLCVB), Tobacco vein clearing virus (TVCV), Bell pepper endornavirus (BPEV). Tomato leaf curl Ranchi virus (ToLCRnV), Tomato leaf curl Bangladesh betasatellite (ToLCBDB), Tomato leaf curl virus (ToLCJoV), Tomato leaf curl Gujarat virus (TolCGV), Cotton leaf curl virus (CLCuV), Croton yellow vein mosaic virus (CYVMVA), Pepper leaf curl virus betasatellite (PepLCVB), Gaillardia latent virus (GaILV). Grape vine: Grapevine rupestris stem pitting-associated virus (GRSPaV), Grapevine virus B (GVB), Grapevine fleck virus (GFkV), Grapevine leafroll-associated virus (GLRaV-3), Hop stunt viroid (HSVd)*.

The full analysis mode of Taxonomer (Flygare et al., 2016) was tested on the two RNA-seq datasets. However, as plant genomes are not included specifically in the built-in *k-mer* databases the majority of the reads were classified as unknown. Only 8% of the pear RNA-seq reads were classified and <1% of the pepper reads. Taxonomer did classify 5,707 reads as virus for pear and 364,959 reads as virus for pepper. Of the 4 viruses identified in the original study for pear, Taxonomer only identified one (ASGV), but of the 13 originally identified in pepper the tool successfully identified 8 (ALPV, PeSV, ChLCuV, TolcRnV, TolcJov, TolcBDB, PepLCBV, PepLCPV). The exclusion of plants as a *k-mer* database for classification influences the results given by this tool. Better results were obtained for pepper, which might reflect the paired-end nature of the RNA-seq data, or the fact that the default *k-mer* size (21) used to create the *k-mer* databases might be more suited to one dataset than the other. However, because this tool is using *k-mer* profiling and matching rather than mapping and assembly it is incredibly quick. It took <10 h to return results for the 9.7 M reads of the pepper transcriptome. These results indicate that as a first automatic screening of RNA-seq data Taxonomer (Flygare et al., 2016) works well. The results can be downloaded as a hierarchical JSON formatted file, the read matches as a text file in which the classified viruses are identified by their NCBI Taxonomic identification numbers, and a summary of matched species as an excel spreadsheet. This enables further analysis of the sequences after initial classification.

VirusDetect (Zheng et al., 2017) was tested on sRNA-seq data from grapevine (713.4M bases), and after file upload it gave results in <4 h. The results consisted of an interactive table that gave the Genbank IDs for the viruses identified that linked directly to Genbank (Benson et al., 2013). VirusDetect (Zheng et al., 2017) identified 11 virus isolates in the grape sRNA-seq data, 5 of which were identified in the original study. The additional isolates result from the identification of one extra virus Prunus necrotic ringspot virus, four extra isolates of GRSPaV and one of GFKV. Again the results and the fast nature of the tools make VirusDetect (Zheng et al., 2017) an excellent first pass screen for virus detection in sRNA-seq data from plants.

## Discussion and future directions

The fact that none of the virus detection tools were used by any of the published studies on the detection of viruses in plants using RNA-seq data is perhaps surprising. Whilst some tools were not available at the time of analyses, many were but were not used. One reason for this is likely to be the fact that majority of the tools use algorithms that are available as standalone applications. When such algorithms are use outside the constraints of a rigid workflow there is much greater flexibility over the parameters and databases that can be used for analysis. In addition, many of the tools described in Table 1 have been developed using human clinical samples (only 3 of the 10 use plants for testing), and whilst some tools allow the host genome to be changed to non-human alternatives, many of the parameters in the pipelines will still have been designed to achieve optimal results for human samples. In addition, some workflows map potential virus sequences to old versions of public databases or custom databases that are no longer updated, meaning potential virus matches would be missed. One final factor that might work against the use of rigid workflows, is that using analysis tools outside of a workflow gives greater control over the types of data files than can saved at each stage of the analyses, which is important for further downstream analysis.

In generic terms the main problems facing those developing tools for virus detection from RNA-seq data are; (a) the upload of large NGS raw read files, (b) computer intensive data processing steps (assembly, mapping and alignment), (c) reliance upon pre-computed custom databases and (d) how to make the tools available. In addition, the problem of identifying novel viruses is not directly addressed by any bioinformatics tool, as it requires iterative rounds of assembly and mapping by a user with expert knowledge of viral genomes. The problem of tool availability is one of the most difficult ones to solve. A webserver is the best option for the non-expert (as exemplified above), but this requires significant computer hardware at the remote site and can be accompanied by difficulties of uploading large files [although this was successfully achieved for Taxonomer (Flygare et al., 2016) and VirusDetect (Zheng et al., 2017)]. Using locally installed software for virus detection means data does not have to be uploaded to a remote site, but requires expertise in software installation and the Linux environment.

The use of web based analytical environments such as Galaxy (Afgan et al., 2016) and Yabi (Hunter et al., 2012) appear to offer a solution, but their real advantage only comes if they are installed locally. Local installation of such environments allows them to be customized with the addition of new tools and programs. But this still means that users require access to local hardware that is capable of running computer intensive analyses. The future may lie with viral diagnostics workflows that use an analytical environment deployed using Cloud computing (Liu et al., 2014). Cloud computing makes the best use of multiple computers to provide on-demand access to hosted resources, with clouds essentially being large server farms that make use of virtualization to provide remote users with a large number of virtual machines (VMs) (a VM is software that emulates the behavior of a separate computer running an operating system) (Shanahan et al., 2014). Cloud computing for analysis of NGS data has already been widely implemented (e.g., Stein, 2010) and has been applied to genomic analysis of legume crops (O'Sullivan and Angra, 2016).

Galaxy (Afgan et al., 2016) has already developed its own integrated solution for cloud computing, known as Galaxy CloudMan (Afgan et al., 2012). This is a cloud manager that allows users to deploy and share an instance of Galaxy on a cloud computing infrastructure using a web browser. Using such an application would make it possible to develop analysis workflows for virus detection using Galaxy; and then allow users access, which means users can use the workflow without the need to have substantial local hardware. However, the problem with many cloud based tools is that they have been developed for use with a single cloud service and such services attract costs. Amazon Web Services (AWS) was the first to offer on demand cloud facilities and many applications are tied to this service, CloudMan (Afgan et al., 2012) being an example. Now that Google and Microsoft are offering cloud computing it is important to develop tools that are capable of using different cloud service providers. In a recent development Multi-Cloud Genome Key (that executes a variant analysis workflow using NGS data) (Elshazly et al., 2017) has been designed to work across resources from different commercial clouds. This software is even capable of executing a workflow using a cluster whose nodes come from different clouds. This novel development will be of importance in the future when both academic and commercial clouds evolve further. To date, virus detection using cloud computing has not been implemented; but could be achieved if fast *k-mer* based methods could be incorporated into an analytical environment such as Galaxy (Afgan et al., 2016). But clearly when such methods are developed, this needs to be done in the context of recent advances such as MC-GenomeKey (Elshazly et al., 2017) to optimize the use of cloud based services.

## Author contributions

SJ: formulated the review and wrote 70% of the review. AB: collated information for the tools reviewed. SM: 10% contribution to writing review. LT: 20% contribution to writing review and coordinated work for review.

### Conflict of interest statement

The authors declare that the research was conducted in the absence of any commercial or financial relationships that could be construed as a potential conflict of interest.

## References

[B1] Afgan E. Baker D. van den Beek M. Blankenberg D. Bouvier D. Cech M.. (2016). The Galaxy platform for accessible, reproducible and collaborative biomedical analyses: 2016 update. Nucleic Acids Res. 44:gkw343. 10.1093/nar/gkw343 27137889 PMC4987906

[B2] Afgan E. Chapman B. Taylor J. (2012). CloudMan as a platform for tool, data, and analysis distribution. BMC Bioinformatics 13:315. 10.1186/1471-2105-13-315 23181507 PMC3556322

[B3] Agarwala R. Aleksandr M. (2011). BMTagger. Available online at: ftp://ftp.ncbi.nlm.nih.gov/pub/agarwala/bmtagger/ (Accessed Sept 1, 2017).

[B4] Altschul S. F. Gish W. Miller W. Myers E. W. Lipman D. J. (1990). Basic local alignment search tool. J. Mol. Biol. 215, 403–410. 10.1016/S0022-2836(05)80360-2 2231712

[B5] Audano P. Vannberg F. (2014). KAnalyze: a fast versatile pipelined *K-mer* toolkit. Bioinformatics 30, 2070–2072. 10.1093/bioinformatics/btu152 24642064 PMC4080738

[B6] Bankevich A. Nurk S. Antipov D. Gurevich A. A. Dvorkin M. Kulikov A. S.. (2012). SPAdes: a new genome assembly algorithm and its applications to single-cell sequencing. J. Comput. Biol. 19, 455–477. 10.1089/cmb.2012.0021 22506599 PMC3342519

[B7] Barrero R. A. Napier K. R. Cunnington J. Liefting L. Keenan S. Frampton R. A.. (2017). An internet-based bioinformatics toolkit for plant biosecurity diagnosis and surveillance of viruses and viroids. BMC Bioinformatics 18:26. 10.1186/s12859-016-1428-4 28077064 PMC5225587

[B8] Benson D. A. Cavanaugh M. Clark K. Karsch-Mizrachi I. Lipman D. J. Ostell J.. (2013). GenBank. Nucleic Acids Res. 41, 36–42. 10.1093/nar/gks1195 23193287 PMC3531190

[B9] Bhaduri A. Qu K. Lee C. S. Ungewickell A. Khavari P. A. (2012). Rapid identification of non-human sequences in high-throughput sequencing datasets. Bioinformatics 28, 1174–1175. 10.1093/bioinformatics/bts100 22377895 PMC3324519

[B10] Boonham N. Kreuze J. Winter S. van der Vlugt R. Bergervoet J. Tomlinson J.. (2014). Methods in virus diagnostics: from ELISA to next generation sequencing. Virus Res. 186, 20–31. 10.1016/j.virusres.2013.12.007 24361981

[B11] Carissimo G. van den Beek M. Vernick K. D. Antoniewski C. (2017). Metavisitor, a suite of galaxy tools for simple and rapid detection and discovery of viruses in deep sequence data. PLoS ONE 12:e0168397. 10.1371/journal.pone.0168397 28045932 PMC5207757

[B12] Clark C. A. Davis J. A. Abad J. A. Cuellar W. J. Fuentes S. Kreuze J. F. (2012). Sweetpotato viruses: 15 years of progress on understanding and managing complex diseases. Plant Dis. 96, 168–185. 10.1094/PDIS-07-11-0550 30731810

[B13] Coetzee B. Freeborough M. J. Maree H. J. Celton J. M. Rees D. J. G. Burger J. T. (2010). Deep sequencing analysis of viruses infecting grapevines: virome of a vineyard. Virology 400, 157–163. 10.1016/j.virol.2010.01.023 20172578

[B14] Da Silva C. Zamperin G. Ferrarini A. Minio A. Dal Molin A. Venturini L. (2013). The high polyphenol content of grapevine cultivar tannat berries is conferred primarily by genes that are not shared with the reference genome. Plant Cell 25, 4777–4788. 10.1105/tpc.113.118810 24319081 PMC3903987

[B15] Elshazly H. Souilmi Y. Tonellato P. J. Wall D. P. Abouelhoda M. (2017). MC-GenomeKey: a multicloud system for the detection and annotation of genomic variants. BMC Bioinformatics 18:49. 10.1186/s12859-016-1454-2 28107819 PMC5248509

[B16] Flygare S. Simmon K. Miller C. Qiao Y. Kennedy B. Di Sera T.. (2016). Taxonomer: an interactive metagenomics analysis portal for universal pathogen detection and host mRNA expression profiling. Genome Biol. 17, 111. 10.1186/s13059-016-0969-1 27224977 PMC4880956

[B17] Grabherr M. G. Haas B. J. Yassour M. Levin J. Z. Thompson D. A. Amit I.. (2011). Full-length transcriptome assembly from RNA-Seq data without a reference genome. Nat. Biotechnol. 29, 644–652. 10.1038/nbt.1883 21572440 PMC3571712

[B18] Hirahata M. Abe T. Tanaka N. Kuwana Y. Shigemoto Y. Miyazaki S.. (2007). Genome information broker for viruses (GIB-V): database for comparative analysis of virus genomes. Nucleic Acids Res. 35, 339–342. 10.1093/nar/gkl1004 17158166 PMC1781101

[B19] Ho T. Tzanetakis I. E. (2014). Development of a virus detection and discovery pipeline using next generation sequencing. Virology 471–473, 54–60. 10.1016/j.virol.2014.09.019 25461531

[B20] Hunter A. A. Macgregor A. B. Szabo T. O. Wellington C. A. Bellgard M. I. (2012). Yabi: an online research environment for grid, high performance and cloud computing. Source Code Biol. Med. 7, 1. 10.1186/1751-0473-7-1 22333270 PMC3298538

[B21] Jo Y. Choi H. Kim S.-M. Kim S.-L. Lee B. C. Cho W. K. (2016). Integrated analyses using RNA-Seq data reveal viral genomes, single nucleotide variations, the phylogenetic relationship, and recombination for Apple stem grooving virus. BMC Genomics 17:579. 10.1186/s12864-016-2994-6 27507588 PMC4977635

[B22] Jo Y. Choi H. Kim S.-M. Kim S.-L. Lee B. C. Cho W. K. (2017). The pepper virome: natural co-infection of diverse viruses and their quasispecies. BMC Genomics 18:453. 10.1186/s12864-017-3838-8 28595635 PMC5465472

[B23] Jo Y. Choi H. Kyong Cho J. Yoon J.-Y. Choi S.-K. Kyong Cho W. (2015). In silico approach to reveal viral populations in grapevine cultivar Tannat using transcriptome data. Sci. Rep. 5:15841. 10.1038/srep15841 26508692 PMC4623741

[B24] Kashif M. Pietilä S. Artola K. Jones R. A. C. Tugume A. K. Makinen V. (2012). Detection of viruses in sweetpotato from honduras and guatemala augmented by deep-sequencing of small-RNAs. Plant Dis. 96, 1430–1437. 10.1094/PDIS-03-12-0268-RE 30727310

[B25] Langmead (2013). Bowtie2. Nat. Methods 9, 357–359. 10.1038/nmeth.1923 PMC3322381 22388286

[B26] Leinonen R. Sugawara H. Shumway M. (2011). The sequence read archive. Nucleic Acids Res. 39, 2010–2012. 10.1093/nar/gkq1019 21062823 PMC3013647

[B27] Li H. Durbin R. (2009). Fast and accurate short read alignment with Burrows-Wheeler transform. Bioinformatics 25, 1754–1760. 10.1093/bioinformatics/btp324 19451168 PMC2705234

[B28] Li H. Ruan J. Durbin R. (2008). Mapping short DNA sequencing reads and calling variants using mapping. Genome Res. 1851–1858. 10.1101/gr.078212.108 18714091 PMC2577856

[B29] Li R. Gao S. Hernandez A. G. Wechter W. P. Fei Z. Ling K. S. (2012). Deep sequencing of small RNAs in tomato for virus and viroid identification and strain differentiation. PLoS ONE 7:e37127. 10.1371/journal.pone.0037127 22623984 PMC3356388

[B30] Li W. Godzik A. (2006). Cd-hit: a fast program for clustering and comparing large sets of protein or nucleotide sequences. Bioinformatics 22, 1658–1659. 10.1093/bioinformatics/btl158 16731699

[B31] Li Y. Wang H. Nie K. Zhang C. Zhang Y. Wang J.. (2016). VIP: an integrated pipeline for metagenomics of virus identification and discovery. Sci. Rep. 6:23774. 10.1038/srep23774 27026381 PMC4824449

[B32] Liu B. Madduri R. K. Sotomayor B. Chard K. Lacinski L. Dave U. J.. (2014). Cloud-based bioinformatics workflow platform for large-scale next-generation sequencing analyses. J. Biomed. Inform. 49, 119–133. 10.1016/j.jbi.2014.01.005 24462600 PMC4203338

[B33] Malhotra S. Sowdhamini R. (2013). Genome-wide survey of DNA-binding proteins in Arabidopsis thaliana: analysis of distribution and functions. Nucleic Acids Res. 41, 7212–7219. 10.1093/nar/gkt505 23775796 PMC3753632

[B34] Marchler-Bauer A. Derbyshire M. K. Gonzales N. R. Lu S. Chitsaz F. Geer L. Y.. (2015). CDD: ncbi's conserved domain database. Nucleic Acids Res. 43, D222–D226. 10.1093/nar/gku1221 25414356 PMC4383992

[B35] Massart S. Candresse T. Gil J. Lacomme C. Predajna L. Ravnikar M.. (2017). A framework for the evaluation of biosecurity, commercial, regulatory, and scientific impacts of plant viruses and viroids identified by NGS technologies. Front. Microbiol. 8:45. 10.3389/fmicb.2017.00045 28174561 PMC5258733

[B36] Matsumura E. Coletta-Filho H. Nouri S. Falk B. Nerva L. Oliveira T.. (2017). Deep sequencing analysis of RNAs from citrus plants grown in a citrus sudden death-affected area reveals diverse known and putative novel viruses. Viruses 9:92. 10.3390/v9040092 28441782 PMC5408698

[B37] Mumford R. Boonham N. Tomlinson J. Barker I. (2006). Advances in molecular phytodiagnostics - new solutions for old problems. Eur. J. Plant Pathol. 116, 1–19. 10.1007/s10658-006-9037-0 PMC7087944 32214677

[B38] O'Leary N. A. Wright M. W. Brister J. R. Ciufo S. Haddad D. McVeigh R.. (2016). Reference sequence (RefSeq) database at NCBI: current status, taxonomic expansion, and functional annotation. Nucleic Acids Res. 44, D733–D745. 10.1093/nar/gkv1189 26553804 PMC4702849

[B39] O'Sullivan D. M. Angra D. (2016). Advances in faba bean genetics and genomics. Front. Genet. 7:150. 10.3389/fgene.2016.00150 27597858 PMC4993074

[B40] Petty T. J. Cordey S. Padioleau I. Docquier M. Turin L. Preynat-Seauve O.. (2014). Comprehensive human virus screening using high-throughput sequencing with a user-friendly representation of bioinformatics analysis: a pilot study. J. Clin. Microbiol. 52, 3351–3361. 10.1128/JCM.01389-14 25009045 PMC4313162

[B41] Pickett B. E. Sadat E. L. Zhang Y. Noronha J. M. Squires R. B. Hunt V.. (2012). ViPR: An open bioinformatics database and analysis resource for virology research. Nucleic Acids Res. 40, 593–598. 10.1093/nar/gkr859 22006842 PMC3245011

[B42] Rampelli S. Soverini M. Turroni S. Quercia S. Biagi E. Brigidi P.. (2016). ViromeScan: a new tool for metagenomic viral community profiling. BMC Genomics 17:165. 10.1186/s12864-016-2446-3 26932765 PMC4774116

[B43] Rybicki E. P. (2015). A top ten list for economically important plant viruses. Arch. Virol. 160, 17–20. 10.1007/s00705-014-2295-9 25430908

[B44] Schulz M. H. Zerbino D. R. Vingron M. Birney E. (2012). Oases: robust *de novo* RNA-seq assembly across the dynamic range of expression levels. Bioinformatics 28, 1086–1092. 10.1093/bioinformatics/bts094 22368243 PMC3324515

[B45] Shanahan H. P. Owen A. M. Harrison A. P. (2014). Bioinformatics on the cloud computing platform Azure. PLoS ONE 9:e102642. 10.1371/journal.pone.0102642 25050811 PMC4106841

[B46] Smit A. Hubley R. Green P. (2013). RepeatMasker Open-4.0. 2013-2015. Available online at: http://www.repeatmasker.org.

[B47] Squires R. B. Noronha J. Hunt V. García-Sastre A. Macken C. Baumgarth N.. (2012). Influenza research database: an integrated bioinformatics resource for influenza research and surveillance. Influenza Other Respir. Viruses 6, 404–416. 10.1111/j.1750-2659.2011.00331.x 22260278 PMC3345175

[B48] Stein L. D. (2010). The case for cloud computing in genome informatics. Genome Biol. 11, 207. 10.1186/gb-2010-11-5-207 20441614 PMC2898083

[B49] Suzek B. E. Wang Y. Huang H. McGarvey P. B. Wu C. H. (2015). UniRef clusters: a comprehensive and scalable alternative for improving sequence similarity searches. Bioinformatics 31, 926–932. 10.1093/bioinformatics/btu739 25398609 PMC4375400

[B50] Syller J. (2012). Facilitative and antagonistic interactions between plant viruses in mixed infections. Mol. Plant Pathol. 13, 2014–2016. 10.1111/j.1364-3703.2011.00734.x 21726401 PMC6638836

[B51] Wang Q. Jia P. Zhao Z. (2013). VirusFinder: software for efficient and accurate detection of viruses and their integration sites in host genomes through next generation sequencing data. PLoS ONE 8:e64465. 10.1371/journal.pone.0064465 23717618 PMC3663743

[B52] Wu Q. Luo Y. Lu R. Lau N. Lai E. C. Li W.-X.. (2010). Virus discovery by deep sequencing and assembly of virus-derived small silencing RNAs. Proc. Natl. Acad. Sci. U.S.A. 107, 1606–1611. 10.1073/pnas.0911353107 20080648 PMC2824396

[B53] Wylie S. J. Li H. Saqib M. Jones M. G. K. (2014). The global trade in fresh produce and the vagility of plant viruses: a case study in garlic. PLoS ONE 9:e105044. 10.1371/journal.pone.0105044 25133543 PMC4136854

[B54] Zerbino D. R. Birney E. (2008). Velvet: algorithms for *de novo* short read assembly using de Bruijn graphs. Genome Res. 18, 821–829. 10.1101/gr.074492.107 18349386 PMC2336801

[B55] Zhao G. Krishnamurthy S. Cai Z. Popov V. L. Travassos da Rosa A. P. Guzman H.. (2013). Identification of novel viruses using virushunter – an automated data analysis pipeline. PLoS ONE 8:e78470. 10.1371/journal.pone.0078470 24167629 PMC3805514

[B56] Zhao Y. Tang H. Ye Y. (2012). RAPSearch2: a fast and memory-efficient protein similarity search tool for next-generation sequencing data. Bioinformatics 28, 125–126. 10.1093/bioinformatics/btr595 22039206 PMC3244761

[B57] Zheng Y. Gao S. Padmanabhan C. Li R. Galvez M. Gutierrez D.. (2017). VirusDetect: an automated pipeline for efficient virus discovery using deep sequencing of small RNAs. Virology 500, 130–138. 10.1016/j.virol.2016.10.017 27825033

